# On Tinnitus Aurium

**Published:** 1887-12

**Authors:** Thomas Barr

**Affiliations:** Glasgow


### ON TINNITUS AURIUM.

BY THOMAS BARR, M. D., GLASGOW.

[Abstract of an Introduction to a Discussion in the Sub-section of Otology at the Annual Meeting of the British Medical Association, held at Dublin, August, 1887.]

It is not my intention to place before you anything like a review of the etiology, pathology and treatment of this symptom, as such a course would lead us over far too wide a field of discussion. I cannot better occupy the time at my disposal than by considering shortly, and inviting the expression of your views upon, a few of the special remedies which are recommended or employed for the relief of this symptom. I exclude from consideration these remedial measures, whether operative or medicinal, which are directed to the removal of definite, well-recognized lesions in the external or middle ear, upon the existence of which the tinnitus may depend, and by the cure of which the tinnitus is fortunately often removed. Excluding the consideration of these or other forms of treatment intended to remove any palpable lesion or any evident pathological source in the naso-pharynx or ear, we shall inquire what other weapons do we possess, in addition to such modes of treatment, which may be efficiently employed for combating this symptom. We cannot, I think, do better than confer with each other as to the relative and actual value of the various means which have been proposed and tried in such intractable forms of tinnitus aurium.

The first of these to which I would beg to direct your attention is *electrical* treatment. Great diversity of opinion seems to prevail as to the value of this mode of treatment in tinnitus. I think that in the use of electricity to the ear the mode of applying the electrodes is of great importance. The plan of placing one electrode over the mastoid process or to the orifice of the ear, and the other in the hand of the opposite side, is not, I think, a good one. As thus applied, I believe it to be less efficient and also more apt to excite giddiness or flashes of light. Any good results I have seen have been achieved by applying one electrode, in the form of a piece of sponge attached to the conducting wire, and placed at the end of a vulcanite speculum, deep into the external auditory canal, even to the outer surface of the tympanic membrane; and the other, by means of a metalic ring at the end of a catheter, to the pharyngeal orifice of the Eustachian tube. In this way the galvanic current is passed, as completely as possible, through the middle ear. My impression is that any benefit derived is obtained more from the action of the electricity upon the middle ear than from its effects upon the internal ear, and that the benefit is mainly connected with its action upon the intrinsic muscles of the middle ear. We know the far-reaching influences of disturbance of the muscular apparatus of the Eustachian tube and tympanum upon the whole organ of hearing—first, upon the tension of the tympanic structures, and, secondly, upon the tension of the labyrinthine fluid, and the more efficiently we act upon these tubal and tympanic muscles the more likely are we to have good results. It is very much to be desired that this method of treatment should be placed upon a firmer and clearer basis than heretofore, and it is possible that the more general use of a galvanometer and rheostat may assist in the attainment of this result.

Another method of treatment to which I would ask your attention is the *Tonbehandlung*, tone treatment, brought recently before the profession by Professor Lucæ, of Berlin. Itard and Urbantschitsch had already pointed out the effects of objective sounds outside the body upon subjective sounds in the ear. Lucæ has found that a striking influence is frequently exerted upon certain subjective sounds in the ear, mainly of a musical character, by bringing to bear upon them objective sounds coming from a tuning fork, if these objective sounds be as far removed as possible in pitch from the subjective ones. For example if the sounds in the ear are high-pitched notes, such as hissing, ringing, whistling, a deep-toned tuning fork, such as C or $C^1$, is employed; on the other hand, if the sound be low pitched, such as rushing, buzzing, humming, or a low-toned bell, then a tuning fork of a high pitch, such as $C^3$ or $C^4$, is used. The vibrating fork is applied either by placing the end of its handle into the external meatus, or, in order to augment the sound, the vibrations are passed through a resonator fixed into the orifice of the ear. The duration of the application may extend from one minute to five minutes, and in order to ensure a continuous sound the tuning fork may be connected with a magneto-electric apparatus. I think Lucæ's observations are of great value, not only in relation to tinnitus aurium, but also to deafness itself. We know that patients frequently assert that the noises in the ear aggravate the deafness, and that if we could banish the sounds the hearing would be improved. Since Lucæ's observations were published I have tried this tone treatment, and I have met with a considerable measure of success, and am of opinion that greater success is in store for this method of treatment. And, as confirming the impressions of patients in regard to the effects upon the hearing, I have found that the hearing power has been distinctly improved, both as tested by watch and speech, during the disappearance or subsidence of the sounds.

Probably of all medicines used for the relief of tinnitus, *hydrobromic acid* has been most extolled. I must confess, gentlemen, my success has not confirmed the panegyrics of some writers. I have prescribed it very frequently indeed, and the somewhat meager results have been disappointing. Our Chairman, whose writings on the pathology and treatment of tinnitus have been so suggestive and interesting, is of opinion that this medicine acts beneficially by bringing about contraction of the dialated and engorged vessels of the labyrinth, through its effects upon the vaso-motor system of the labyrinth. I am afraid, ,however, that in the forms of tinnitus I am now describing we have generally to do not so much with dilated and engorged vessels, but with the more serious condition of pressure exercised upon the nerve or labyrinthine fluid by condensed, thickened, rigid, or morbid tissue. Whether hydrobromic acid has the power of contracting dilated vessels in the labyrinth is not easy to prove. I can only say that in my experience it has only in a very few cases had the effect of removing or materially mitigating noises in the ear. This experience agrees with that of Politzer, expressed in his "Text-book of Disease of the Ear."

The *bromides*, especially bromide of potassium, have been, in my practice, more serviceable than hydrobromic acid. In doses of from half a drachm to a drachm at bed-time, bromide of potassium has not infrequently a distinctly mitigating influence upon the noises.

*Pilocarpine*, employed hypodermically, is undoubtedly useful in a certain proportion of cases. In my hands this remedy has been strikingly successful in several cases, although, alas! like other remedies for the relief of tinnitus aurium, it often fails. I believe it is only useful in recent and minute hæmorrhages into the cavities of the labryrinth, but fails if the hæmorrhage is extensive—because in this case, as might be expected, the delicate structures of the labyrinth are destroyed beyond the power of recovery, even after the effused blood has become absorbed. As to the explanation of the therapeutic action of pilocarpine, we can only assume that it has an especial power of stimulating the absorbents in contact with the effused products before these have become organized, and that this resorbent effect has also some connection with its remarkable powers of exciting the cutaneous and salivary secretion. It seems to have an especial action upon the intracranial absorbents, and we know that the vascular and lymphatic supply of the labyrinth is in reality the same as that of the interior of the cranium.

We are all familiar with the fact that *quinine* has an influence upon the circulation of the ear, especially of the internal ear, and that noises in the ear with impairment of hearing are frequently set up by this drug. There can be no doubt, however, that in the cases I am referring to large doses of quininine (5 to 20 grains)not infrequently exercise a beneficial effect. How this is brought about it is not easy to say ; further and careful clinical observations are required before a decision can be arrived at in regard to the exact position of quinine as a remedy in these forms of tinnitus aurium.

I do not think that it would be profitable or interesting if I were to discuss the merits of all the multifarious medicinal remedies proposed and used for the relief of noises in the ear. Unfortunately, the weapons taken from this well-stocked therapeutic armory are too often feeble and inefficient.

On the merits of nitrite of amyl, strychnine, arsenic, digitals, nitro-glycerine, ergotine, convallaria, chloride of ammonium, etc., we need not descant. I trust that out of the wealth of your experience we may to-day add to our knowledge of the true position of some of these remedies. Neither need I point out the necessity of employing appropriate hygienic and dietetic treatment, if any departure from the healthy condition is manifest; nor of the importance of mercurials, laxatives, and salines, if the hepatic or stomachic functions are disturbed; nor of the value of ferruginous tonics in anæmia, and anti-syphilitic remedies where a specific origin is clearly traced. These considerations are so self-evident that their discussion by me would be quite superfluous.—*British Medical Journal.*

